# Clinical Characteristics of COVID-19 Patients and Application to an Artificial Intelligence System for Disease Surveillance

**DOI:** 10.3390/jcm11051437

**Published:** 2022-03-05

**Authors:** Ying-Chuan Wang, Dung-Jang Tsai, Li-Chen Yen, Ya-Hsin Yao, Tsung-Ta Chiang, Chun-Hsiang Chiu, Te-Yu Lin, Kuo-Ming Yeh, Feng-Yee Chang

**Affiliations:** 1 Department of Family Medicine, Tri-Service General Hospital, National Defense Medical Center, Taipei 11499, Taiwan; popoga96@gmail.com; 2 Graduate Institute of Life Sciences, National Defense Medical Center, Taipei 11499, Taiwan; oo800217@gmail.com; 3 School of Public Health, National Defense Medical Center, Taipei 11499, Taiwan; 4 Department of Microbiology and Immunology, National Defense Medical Center, Taipei 11499, Taiwan; yenlichen1030@gmail.com; 5 National Defense Medical Center, Taipei 11499, Taiwan; yamcom13579@gmail.com; 6 Division of Infectious Diseases and Tropical Medicine, Department of Internal Medicine, Tri-Service General Hospital, National Defense Medical Center, Taipei 11499, Taiwan; cgdada@gmail.com (T.-T.C.); lin.deyu@msa.hinet.net (T.-Y.L.); kmyeh@mail.ndmctsgh.edu.tw (K.-M.Y.); fychang@mail.ndmctsgh.edu.tw (F.-Y.C.)

**Keywords:** COVID-19, artificial intelligence, support vector machine (SVM), decision tree, random forest, artificial neural network

## Abstract

During the coronavirus disease (COVID-19) pandemic, we admitted suspected or confirmed COVID-19 patients to our isolation wards between 2 March 2020 and 4 May 2020, following a well-designed and efficient assessment protocol. We included 217 patients suspected of COVID-19, of which 27 had confirmed COVID-19. The clinical characteristics of these patients were used to train artificial intelligence (AI) models such as support vector machine (SVM), decision tree, random forest, and artificial neural network for diagnosing COVID-19. When analyzing the performance of the models, SVM showed the highest sensitivity (SVM vs. decision tree vs. random forest vs. artificial neural network: 100% vs. 42.86% vs. 28.57% vs. 71.43%), while decision tree and random forest had the highest specificity (SVM vs. decision tree vs. random forest vs. artificial neural network: 88.37% vs. 100% vs. 100% vs. 94.74%) in the diagnosis of COVID-19. With the aid of AI models, physicians may identify COVID-19 patients earlier, even with few baseline data available, and segregate infected patients earlier to avoid hospital cluster infections and to ensure the safety of medical professionals and ordinary patients in the hospital.

## 1. Introduction

Coronavirus disease 2019 (COVID-19) is an ongoing global pandemic caused by the severe acute respiratory syndrome coronavirus 2 (SARS-CoV-2). The clinical spectrum of COVID-19 appears to be broad, ranging from no symptoms to mild upper respiratory tract illness, severe pneumonia with respiratory failure, and death. The existence of asymptomatic patients and patients with non-specific symptoms may significantly delay the diagnosis of COVID-19 and present a serious threat to public health. The rising incidence and massive casualties of COVID-19 exert significant pressure on limited healthcare resources. The early diagnosis of asymptomatic or mild COVID-19 patients is essential to prevent the spread of the infection during the pandemic. However, the gold standard for COVID-19 diagnosis, the reverse transcriptase polymerase chain reaction (RT-PCR), takes a maximum of up to two days to give the result. There has also been a heavy shortage of RT-PCR test kits in many countries during the pandemic. Thus, effective tools are really needed to simplify the diagnosis and surveillance of COVID-19. Recently, researchers found that well-trained artificial intelligence (AI) can ensure accurate and rapid diagnosis or assist physicians to reduce manual labor. Some of these studies were conducted for AI-assisted COVID-19 diagnosis [1,2,3,4,5,6,7,8,9,10,11,12,13,14,15,16,17,18], some were conducted for predicting the prognosis of patients [19,20,21,22,23,24,25,26,27,28], and others were conducted for predicting the epidemic trend of COVID-19 [29,30,31].

Taiwan was initially expected to be one of the most affected countries owing to its geographic proximity and close people-to-people exchanges with China [32]. However, as the disease continues to spread globally, Taiwan has been able to contain the pandemic and minimize its impact on the daily lives of its citizens. Since the first confirmed case in Taiwan on 21 February 2020 [33], less than 1100 cases were reported in Taiwan till 10 April 2021. This is attributed to the government’s rapid action including border control from the air and sea, adequate screening, quarantine of suspicious cases, identification of travelers’ infection risks, and comprehensive contact tracing. To coordinate the pandemic-prevention policies, our hospital set up two isolation wards to admit confirmed COVID-19 patients and suspected cases since 2 March 2020. Until 4 May 2020, 217 patients had been hospitalized into our isolation ward, of which 27 had a confirmed COVID-19 diagnosis. To facilitate diagnosing COVID-19, we tried to apply the clinical characteristics of these patients to different AI models and find the most effective one.

There are limited data on the clinical characteristics of COVID-19 patients in Taiwan. We aimed to delineate the epidemic prevention experience of our hospital under the guidance of Taiwan’s government, clarify the differences in clinical characteristics between confirmed cases and COVID-19-negative patients admitted to our hospital, and apply the clinical characteristics to AI models for diagnosing COVID-19.

## 2. Materials and Methods

### 2.1. Study Population

Our study included adult patients (age ≥ 20 years) with suspected or confirmed COVID-19 diagnosis, who were admitted to our isolation wards between 2 March 2020 and 4 May 2020. All patients with COVID-19 were confirmed by using real-time reverse-transcriptase polymerase chain reaction (RT-PCR) assays from oropharyngeal swab specimens. The patients included travelers entering Taiwan with a positive COVID-19 test performed at the airport, symptomatic patients with a contact or travel history who visited our emergency room and needed hospitalization, and people with close contact with confirmed cases and who needed hospitalization. The study was approved by the Institutional Review Board of Tri Service General Hospital, and informed consent was obtained from all patients.

### 2.2. COVID-19 Screening, Hospitalization, and Home Quarantine

**Figure 1:**
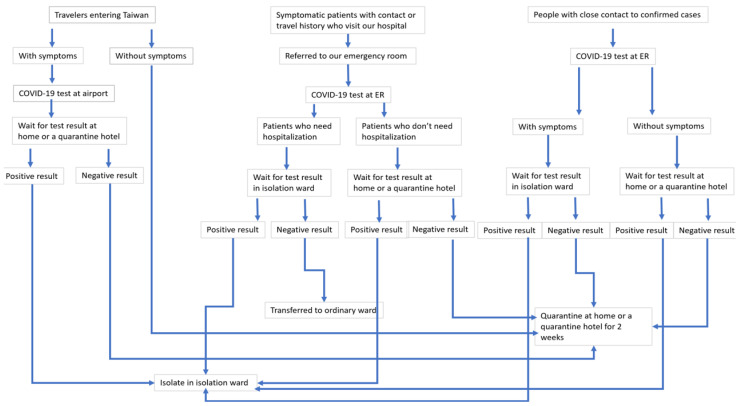
Flow diagram of COVID-19 screening, hospitalization, and home quarantine for travelers entering Taiwan, symptomatic patients who visited our emergency room, and people with close contact with confirmed cases.

Figure 1 shows a flow diagram of the protocols followed for COVID-19 screening, hospitalization, and home quarantine. All travelers entering Taiwan were required to stay at home or at a quarantine hotel and undergo home quarantine for two weeks. Among them, individuals with symptoms were tested at the airport for SARS-CoV-2 viral nucleic acid using RT-PCR assays from oropharyngeal swab specimens. They had to stay at home or at the quarantine hotel while awaiting the test results. Individuals with a positive result in the COVID-19 test were sent to the appointed hospital for isolation and treatment. People with a negative result continued the two-week home quarantine. Symptomatic patients who visited our hospital with a travel or contact history were referred to our emergency room (ER) and tested for COVID-19. Patients who needed hospitalization were admitted to our isolation ward while waiting for the test result. Among them, patients with a positive result remained hospitalized in the isolation ward, while patients with a negative result were transferred to an ordinary ward. Patients in the ER who did not need hospitalization were asked to stay at home until the test results. Among them, patients with a positive result were admitted to the isolation ward, while patients with a negative result home-quarantined for two weeks. Individuals with close contact with confirmed cases were sent to the appointed hospital for COVID-19 testing, and symptomatic people among them were isolated in the dedicated ward until the results arrived. People without symptoms stayed at home or at a quarantine hotel while waiting for the result. Among them, people with positive results were arranged hospitalization in the isolation ward, while COVID-19-negative patients continued the home quarantine for two weeks. All transportation between the airport, home, the quarantine hotel, and the hospital were made through appointed cars instead of public transportation.

### 2.3. Obtaining the Demographic Data, Clinical Symptoms, and Laboratory Data

Relevant clinical data of the enrolled people, including age, gender, underlying diseases, clinical symptoms, and laboratory data, were recorded. Laboratory data including white blood cell count, platelet count, neutrophil-to-lymphocyte ratio, renal function, liver function, levels of total bilirubin, C-reactive protein, D-dimer, and procalcitonin were examined and noted within 24 h after admission. Patients were diagnosed with pneumonia based on the lower respiratory tract symptom of cough, the systemic symptom of fever, and new onset radiology findings of infiltration [34,35].

### 2.4. Statistical Analysis

The patients were sub-grouped in confirmed COVID-19 patients and suspected cases with a negative result to compare their clinical characteristics, including demographic data, underlying diseases, symptoms, and laboratory data. All results were analyzed using a commercially available software package (SPSS, version 21.0; SPSS Inc., Chicago, IL, USA). Categorical variables were analyzed using the chi-square test, while continuous variables with categorical variables were analyzed with the independent two-samples *t* test for comparison. All *p*-values were 2-tailed, and *p*-values of less than 0.05 were considered to indicate statistical significance.

### 2.5. Applying the Clinical Characteristics and Routine Laboratory Data to Train AI Models

**Figure 2:**
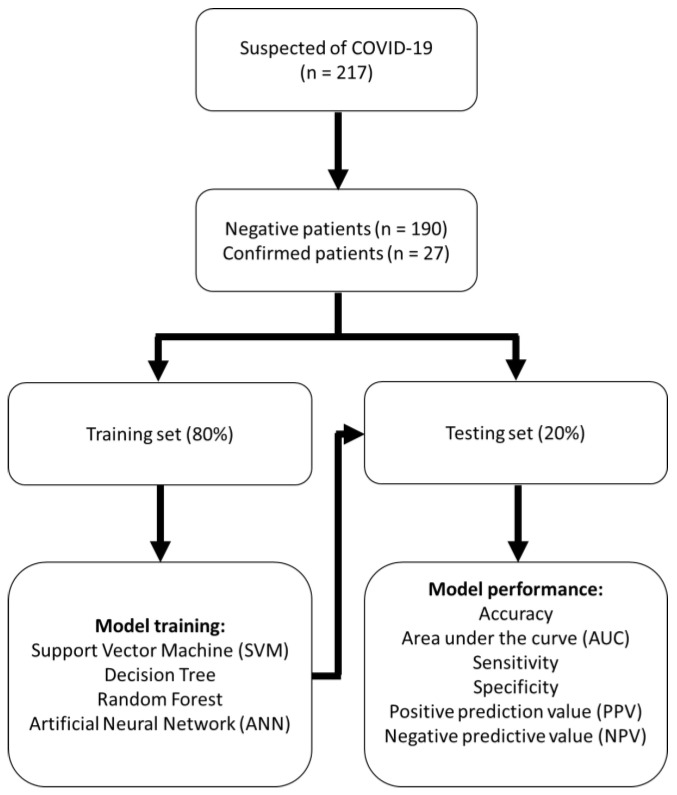
Flow chart of patients included in the model building and validation process.

In order to obtain a confirmed COVID-19 predictive model (“Outcome” feature), we established four AI models including support vector machine (SVM), decision tree, random forest, and artificial neural network by inputting the above information comprising clinical characteristics (sex, age, temperature, SBP, DBP, PR, RR, fever, cough, headache, muscle ache, distorted sense of taste, distorted sense of smell, rhinorrhea, sore throat, chest tightness, dyspnea, diarrhea, eye illness, nausea and vomiting) and routine laboratory data (WBC/1000, PLT, Neu(%), ANC, Lym(%), ALC, Cr, CRP, AST, ALT). We created training and testing sets by splitting the sample randomly to assess the performance of the model. A classifier can only be trained using retrospective data in the real world, and it will be used to classify future data. The machine learning construction process was to split all data into training and test datasets using 80% and 20% of the data. The process is shown in Figure 2.

#### 2.5.1. Support Vector Machines

Support vector machines (SVMs) are common classifiers in machine learning. They map all samples to a hyperplane and separate them with a clear space. In addition, core tips are used to extend this hyperplane. SVMs have been shown to perform better in classifying free-text medical literature than naive Bayesian classifiers, C4.5 decision trees, and adaptive amplification [36]. In this study, we used the four most common kernel tips: linear, polynomial (degree = 3), radial basis, and sigmoid. We used the e1071 package (R package version 1.7-4) as the SVM implementation and set all other parameters to their defaults [37].

#### 2.5.2. Random Forest

A random forest (RF) generates multiple decision trees and uses information from each tree to make predictions. This is the best classification model in previous text classification research [38] compared with SVM, Bayes classifier, and k-nearest neighbor algorithm. We used the version package 4.6-14 [39] as the RF implementation and set all the parameters to their default values.

#### 2.5.3. Decision Tree

A decision tree is a non-parametric method among the supervised learning methods. Supervised learning means automatically building predictive models via algorithms from a given set of observations (data) as a training dataset [40]. Test datasets are used to assess how good the algorithm predicts the outcome from unseen data, which is also known as model evaluation. For decision tree analysis, the variables do not need to be linear/normal or additive, and their possible interactions do not need to be pre-specified. Missing values of the covariates, multicollinearity, and outliers are automatically taken into account [41]. We used the party package (R package version 1.3-6) [42] as the decision tree implementation and set all other parameters to their default values.

#### 2.5.4. Artificial Neural Network

An Artificial Neural Network is a computational model inspired by the functioning of the human brain. It is composed by a set of artificial neurons (known as processing units) that are interconnected with other neurons. Each connection has an associated weight that represents the influence of one neuron on another. The word network in Neural Network refers to the interconnection between neurons present in various layers of a system. Every system is basically a 3-layered system, and the layers are the Input layer, the Hidden Layer, and the Output Layer. The input layer has input neurons which transfer data via synapses to the hidden layer, and similarly the hidden layer transfers these data to the output layer via more synapses. The synapses store values called weights which help them to manipulate the input and output to various layers. An ANN can be defined based on the following three characteristics:

1. The architecture indicating the number of layers and the number of nodes in each layer.
2. The learning mechanism applied for updating the weights of the connections.
3. The activation functions used in various layers.
  We used the MXNet version 0.8.0 package [43] to implement the above architecture. The settings used for the training model were as follows: (1) the network architecture was 4 × 3 × 1, i.e., the input layer had 4 nodes, the hidden layer had 3 nodes, and the output layer had 1 node; (2) minibatch gradient descent with batch size of 20 for optimization; (3) learning rate = 0.013; (4) momentum coefficient = 0.9; (4) L2 regularization coefficient = 0.

## 3. Results

From 2 March 2020 to 4 May 2020, there were 217 cases suspected of COVID-19 who were admitted to our isolation ward at Tri Service General Hospital. The median patient age was 40.8 years (range, 1–92 years). Among them, 107 (49.3%) were male, 101 (46.5%) had pneumonia, while 27 (12.4%) were finally confirmed to have contracted COVID-19.

### 3.1. Demographic Data and Underlying Diseases of Confirmed COVID-19 Patients and COVID-19-Negative Patients

The demographic data and underlying diseases of confirmed COVID-19 patients and COVID-19-negative patients are listed in Table 1. There was no gender predominance in both the confirmed group (male vs. female; 51.9% vs. 48.1%) and the negative group (male vs. female; 48.9% vs. 51.1%). The median age of the confirmed patients was 41.7 ± 18.5 years, while that of the negative patients was 40.7 ± 20.4 years. The confirmed COVID-19 patients had a higher prevalence of hyperlipidemia than the COVID-19-negative patients (18.5% vs. 2.6%; *p* < 0.001). Between the two groups, there was no significant difference in the prevalence of hypertension, diabetes mellitus, hyperuricemia, chronic kidney disease, cerebrovascular accident, coronary artery disease, cardiac arrhythmia, valvular heart disease, congestive heart failure, bronchial asthma, chronic obstructive pulmonary disease, solid organ cancer, hematogenic disorder, human immunodeficiency virus infection, chronic hepatitis, auto-immune disease, chronic urticaria, or allergic rhinitis

### 3.2. Symptoms of Confirmed COVID-19 and COVID-19-Negative Patients

The symptoms of confirmed COVID-19 and COVID-19-negative patients are listed in Table 2. The most frequent symptoms in both groups were cough and fever (confirmed cases vs. negative cases; 81.5% vs. 52.1% and 63% vs. 43.7%, respectively). The confirmed COVID-19 patients, compared to the negative patients, had a higher prevalence of cough (81.5% vs. 52.1%; *p* = 0.004), distorted sense of taste (25.9% vs. 0; *p* < 0.001), distorted sense of smell (37% vs. 0.5%; *p* < 0.001), rhinorrhea (44.4% vs. 14.2%; *p* < 0.001), chest tightness (18.5% vs. 6.3%; *p* = 0.027), dyspnea (37% vs. 12.6%; *p* = 0.001), diarrhea (33.3% vs. 5.3%; *p* < 0.001), and nausea and vomiting (11.1% vs. 2.1%; *p* = 0.013). On the contrary, there was no significant difference in the prevalence of fever, headache, muscle ache, sore throat, or eye illness.

### 3.3. Laboratory and Radiological Findings of Confirmed COVID-19 Patients and COVID-19-Negative Cases

The laboratory and radiological findings of confirmed COVID-19 patients and COVID-19-negative cases on admissions are listed in Table 3. Confirmed COVID-19 patients had a lower absolute neutrophil count (3436.7 ± 1151.8 cells/μL vs. 7011.1 ± 8888.9 cells/μL; *p* = 0.038) and a lower absolute lymphocyte count (1334.4 ± 645.5 cells/μL vs. 1912.4 ± 1357.8 cells/μL; *p* = 0.031) than COVID-19-negative cases. Among them, 17 (63%) confirmed COVID-19 patients and 84 (44.2%) COVID-19-negative patients had pneumonia.

### 3.4. Accuracy, Sensitivity, and Specificity of Support Vector Machine (SVM), Decision Tree, Random Forest, and Artificial Neural Network for COVID-19 Detection and Diagnosis

The accuracy, sensitivity, and specificity of the AI models we used for COVID-19 detection and diagnosis are shown in Table 4. In the performance of the models, SVM showed the highest sensitivity (SVM vs. decision tree vs. random forest vs. artificial neural network: 100% vs. 42.86% vs. 28.57% vs. 71.43%), while decision tree and random forest had the highest specificity (SVM vs. decision tree vs. random forest vs. artificial neural network: 88.37% vs. 100% vs. 100% vs. 94.74%).

## 4. Discussion

COVID-19 spread worldwide in just two months since December 2019. Taiwan has been containing it thanks to efforts in early pre-assessment and appraisal to control the disease risk. Between March 2 2020 and May 4 2020, 217 patients with possible COVID-19 were admitted to our isolation wards, with 27 confirmed cases. These 27 confirmed patients recovered and were discharged, with no occurrence of hospital outbreak.

According to the Taiwan Centers for Disease Control and Prevention (CDC), 934 (89.0%) of the 1050 confirmed COVID-19 cases in Taiwan were imported. Thus, travelers entering Taiwan and symptomatic patients with a travel history are thought to be at the highest risk of SARS-CoV-2 infection. A previous study revealed that the real-time effective reproduction number (R(t)) of SARS-CoV-2 was 3.27 for Italy, 6.32 for France, 6.07 for Germany, and 5.08 for Spain [5]. With the time-dependent method, the R(t) value was 3.1 for Italy, 6.56 for France, 4.43 for Germany, and 3.95 for Spain [44]. Owing to the highly contagious nature of SARS-CoV-2, people with close contact with confirmed patients are also considered to be at the highest risk. Thus, physicians in our hospital are requested to be aware of high-risk groups and/or individuals, i.e., travelers entering Taiwan, symptomatic patients with a travel or contact history, and people with close contact with confirmed patients, and are requested to follow the well-designed and efficient assessment protocol of COVID-19 screening, hospitalization, and home quarantine presented in Figure 1. The timely identification of individuals at risk may be one of the main factors that assisted Taiwan in containing the pandemic and in preventing outbreaks in its hospitals.

In our study, confirmed COVID-19 patients had a higher prevalence of hyperlipidemia. According to a New York-based study, the most common comorbidities in COVID-19 fatalities were hypertension (55.4%), diabetes (37.3%), hyperlipidemia (18.5%), and coronary artery disease (12.4%) [45]. In another study conducted in Wuhan, of the 138 patients hospitalized for COVID-19 and requiring an intensive care support, 25% had cardiovascular disease, and 58% had hypertension. Of those who did not require an intensive care units support, 10% had cardiovascular disease and 22% had hypertension [46]. According to these studies and our study, dysfunction of lipid metabolism, associated metabolic dysfunction, or related complications such as atherosclerotic disease may increase the vulnerability or severity of COVID-19. Further studies are needed to clarify the relationship between lipid metabolism and COVID-19 pathophysiology.

Similar to several previous studies, we noted cough and fever to be the most common symptoms in confirmed COVID-19 patients [46,47,48,49]. In contrast to the what observed for severe acute respiratory syndrome (SARS) of 2003, fever is not considered an important indicator for SARS-CoV-2 transmission. Several studies documented SARS-CoV-2 transmission during the pre-symptomatic incubation period [50,51,52,53], while others documented SARS-CoV-2 infection in patients who never developed symptoms (asymptomatic) [54,55,56]. As shown in an increasing number of studies that report a high prevalence of distorted sense of taste or smell in COVID-19 patients [57,58], 37% of our confirmed COVID-19 patients had a distorted sense of smell, and 25.9% had a distorted sense of taste. This could be due to the high expression level of angiotensin-converting enzyme 2 (ACE2) proteins in nasal respiratory epithelial cells and olfactory epithelial support cells [59]. A previous study suggested that the loss of taste and smell, in combination, is a strong predictor of SARS-CoV-2 infection [60]. Physicians should be on alert when patients have distorted smell or taste.

Considering the laboratory findings noted in our study, confirmed COVID-19 patients had a higher prevalence of neutropenia or lymphopenia than COVID-19-negative patients. According to previous studies, lymphopenia was found to be the most common laboratory finding in COVID-19 patients [48,61,62]. Several factors may contribute to COVID-19-related lymphopenia. First, lymphocytes express the ACE2 receptor on their surface and may be a direct target of SARS-CoV-2 [63]. Second, the subsequent cytokine storm with increased expression of interleukin-6 (IL-6), granulocyte colony-stimulating factor (GCSF), tumor necrosis factor (TNF)-α, and other pro-inflammatory cytokines may cause lymphocyte dysfunction or apoptosis [64,65]. Third, the substantial cytokine storm may also be associated with a dysfunction of lymphoid organs such as the spleen [66]. Lymphopenia is considered one of the predictive factors of severe disease in COVID-19 patients [67,68]. Physicians should be vigilant for the presence of lymphopenia in undiagnosed or confirmed COVID-19 patients.

Early detection and timely diagnosis of COVID-19 infections is very helpful to reduce the spread of the virus. However, the nonspecific clinical characteristics of COVID-19 infections make the diagnosis even more difficult. Thus, the development of AI tools for a timely diagnosis of COVID-19 infection is important and imperative, especially in the circumstances when we only have few data regarding suspicious cases. During the pandemic, many AI models were developed for the early detection of COVID-19. Among them, models based on chest computed tomography (CT) images were the most abundant [1,2,3,4,5,6,7,8,9,10,11,12,13,14,15,16,17,18]. Several studies have developed AI techniques to detect and identify features from chest CT images to assist in the diagnosis of COVID-19 with high accuracy (70.00 to 99.87%), sensitivity (73.00 to 100.00%), specificity (25 to 100.00%), and AUC (0.732 to 1.000) [1,2,3,4,5,6,7,8,9,10,11,12,13,14,15,16,17,18]. However, performing CT scan in all suspected COVID-19 patients may cause significant pressure in countries with limited healthcare resources during the pandemic. In our study, we describe a model to early detect COVID-19 infection by inputting clinical characteristics and routine lab data which is more feasible and economic than routine expensive examinations such as chest CT. With the aid of the AI models we developed, we can identify patients at risk of COVID-19 and early decide who should be quarantined and undergo further exams such as chest CT or RT-PCR.

However, the present study has several limitations that must be considered. First, we enrolled only 217 suspected COVID-19 patients in our study. The small sample size may affect the reliability of the results because it leads to a higher variability, which may cause bias. Second, the study patients were enrolled almost 2 years ago. The sensitivity and specificity of the AI models we used to identify COVID-19 maybe different when applying to current diagnosed patients. Third, these AI models may help physicians to identify symptomatic COVID-19 patients earlier by analyzing their clinical characteristics. However, asymptomatic patients may not be identified by using our AI systems. Forth, our study was conducted during the COVID-19 pandemic. As we know, behavioral changes (social distancing, mask wearing, and hygiene measures) and travel and movement restrictions during the COVID-19 pandemic have led to a reduction in the incidence of influenza and other common respiratory infections [69,70]. The high sensitivity and specificity of the AI models we used to identify COVID-19 maybe due to the reduction in the incidence of other common respiratory infections. Further AI-assisted detection tools still need to be studied and developed. 

## 5. Conclusions

In conclusion, COVID-19 is a highly contagious disease characterized by a long period of communicability, varied presentations, and nonspecific laboratory findings. Physicians should be aware of the clinical characteristics of the disease and keenly observe patients at high risk. Besides, the AI-assisted system for the early detection and timely diagnosis of COVID-19 infection needs to be further developed.

## Figures and Tables

**Table 1:** Demographic data and underlying diseases of confirmed COVID-19 patients and COVID-19-negative patients.

		Confirmed Patients	Negative Patients	
Sex	male	14 (51.9%)	93 (48.9%)	*p* = 0.778
	female	13 (48.1%)	97 (51.1%)	
Age (years)		41.7 ± 18.5	40.7 ± 20.4	*p* = 0.801
Underlying diseases				
HTN	yes	3 (11.1%)	32 (16.8%)	*p* = 0.449
	no	24 (88.9%)	158 (83.2%)	
DM	yes	1 (3.7%)	15 (7.9%)	*p* = 0.436
	no	26 (96.3%)	175 (92.1%)	
Hyperlipidemia	yes	5 (18.5%)	5 (2.6%)	*p* < 0.001
	no	22 (81.5%)	185 (97.4%)	
Hyperuricemia	yes	1 (3.7%)	2 (1.1%)	*p* = 0.27
	no	26 (96.3%)	188 (98.9%)	
CKD	yes	0	2 (1.1%)	*p* = 0.592
	no	27(100%)	188 (98.9%)	
CVA	yes	1 (3.7%)	2 (1.1%)	*p* = 0.27
	no	26 (96.3%)	188 (98.9%)	
CAD	yes	0	7(3.7%)	*p* = 0.311
	no	27 (100%)	183 (96.3%)	
Cardiac arrhythmia	yes	0	3 (1.6%)	*p* = 0.511
	no	27 (100%)	187 (98.4%)	
VHD	yes	0	4(2.1%)	*p* = 0.447
	no	27 (100%)	186 (97.9%)	
CHF	yes	0	8 (4.2%)	*p* = 0.447
	no	27 (100%)	182 (95.8%)	
Bronchial asthma	yes	0	7 (3.7%)	*p* = 0.311
	no	27 (100%)	183 (96.3%)	
COPD	yes	0	2 (1.1%)	*p* = 0.592
	no	27 (100%)	188 (98.9%)	
Solid organ cancer	yes	1 (3.7%)	5 (2.6%)	*p* = 0.751
	no	26 (96.3%)	185 (97.4%)	
Hematogenic disorder	yes	0	2 (1.1%)	*p* = 0.592
	no	27 (100%)	188 (98.9%)	
HIV infection	yes	0	2 (1.1%)	*p* = 0.592
	no	27 (100%)	188 (98.9%)	
Chronic hepatitis	yes	2 (7.4%)	5 (2.6%)	*p* = 0.189
	no	25 (92.6%)	185 (97.4%)	
Autoimmune disease	yes	0	5 (2.6%)	*p* = 0.394
	no	27 (100%)	185 (97.4%)	
Chronic urticaria	yes	0	3 (1.6%)	*p* = 0.511
	no	27 (100%)	187 (98.4%)	
Allergic rhinitis	yes	1 (3.7%)	2 (1.1%)	*p* = 0.27
	no	26 (96.3%)	188 (98.9%)	

CAD, coronary artery disease; CHF, congestive heart failure; CKD, chronic kidney disease; COPD, chronic obstructive pulmonary disease; COVID-19, coronavirus disease; CVA, cerebrovascular accident; DM, diabetes mellitus; HIV, human immunodeficiency virus; HTN, hypertension; VHD, valvular heart disease.

**Table 2:** Symptoms of confirmed COVID-19 patients and COVID-19-negative patients.

Symptoms		Confirmed Patients	Negative Patients	
Fever	yes	17 (63%)	83 (43.7%)	*p* = 0.06
	no	10 (37%)	107 (56.3%)	
Cough	yes	22 (81.5%)	99 (52.1%)	*p* = 0.004
	no	5 (18.5%)	91 (47.9%)	
Headache	yes	4 (14.8%)	19 (10%)	*p* = 0.447
	no	23 (85.2%)	171 (90%)	
Muscle ache	yes	5 (18.5%)	15 (7.9%)	*p* = 0.074
	no	22 (81.5%)	175 (92.1%)	
Distorted sense of taste	yes	7 (25.9%)	0	*p* < 0.001
	no	20 (74.1%)	190 (100%)	
Distorted sense of smell	yes	10 (37%)	1 (0.5%)	*p* < 0.001
	no	17 (63%)	189 (99.5%)	
Rhinorrhea	yes	12 (44.4%)	27 (14.2%)	*p* < 0.001
	no	15 (55.6%)	163 (85.8%)	
Sore throat	yes	8 (29.6%)	32 (16.8%)	*p* = 0.109
	no	19 (70.4%)	158 (83.2%)	
Chest tightness	yes	5 (18.5%)	12 (6.3%)	*p* = 0.027
	no	22 (81.5%)	178 (93.7%)	
Dyspnea	yes	10 (37%)	24 (12.6%)	*p* = 0.001
	no	17 (63%)	166 (87.4%)	
Diarrhea	yes	9 (33.3%)	10 (5.3%)	*p* < 0.001
	no	18 (66.7%)	180 (94.7%)	
Eye illness	yes	1 (3.7%)	1 (0.5%)	*p* = 0.106
	no	26 (96.3%)	189 (99.5%)	
Nausea and vomiting	yes	3 (11.1%)	4 (2.1%)	*p* = 0.013
	no	24 (88.9%)	186 (97.9%)	

COVID-19, coronavirus disease 2019.

**Table 3:** Laboratory and radiological findings of confirmed COVID-19 patients and COVID-19-negative patients on admission.

		Confirmed Patients	Negative Patients	
Lab	WBC (/μΛ)	5239 ± 1498	9907 ± 13,371	*p* = 0.072
	Neutrophil (%)	65.4 ± 11.4	68.6 ± 14.3	*p* = 0.27
	ANC (/μL)	3436.7 ± 1151.8	7011.1 ± 8888.9	*p* = 0.038
	Lymphocyte (%)	25.5 ± 11.1	23 ± 12.7	*p* = 0.332
	ALC (/μL)	1334.4 ± 645.5	1912.4 ± 1357.8	*p* = 0.031
	CRP (mg/dL)	1.8 ± 3.1	3.1 ± 6.1	*p* = 0.117
	PCT (ng/mL)	0.08 ± 0.11	0.55 ± 0.84	*p* = 0.071
	D-dimer (mg/L)	0.85 ± 1.8	4.1 ± 8.1	*p* = 0.089
	AST (U/L)	21.1 ± 7.5	26.8 ± 31.8	*p* = 0.353
	ALT (U/L)	18.6 ± 8.6	27.4 ± 37.3	*p* = 0.242
	Total bilirubin (mg/dL)	0.53 ± 0.24	1.01 ± 1.50	*p* = 0.2
	BUN (mg/dL)	13.2 ± 8.1	13.6 ± 9.0	*p* = 0.84
	Cr (mg/dL)	0.82 ± 0.3	0.96 ± 1.26	*p* = 0.57
Pneumonia	yes	17 (63%)	84 (44.2%)	
	no	10 (37%)	106 (55.8%)	*p* = 0.068

AST, aspartate aminotransferase; ALT, alanine aminotransferase; ANC, absolute neutrophil count; ALC, absolute lymphocyte count; BUN, blood urea nitrogen; COVID-19, coronavirus disease 2019; Cr, creatinine; CRP, C-reactive protein; PCT: procalcitonin; WBC, white blood cell.

**Table 4:** Accuracy, area under the curve (AUC), sensitivity, specificity, positive prediction value (PPV), and negative predictive value (NPV) of support Vector Machine (SVM), decision tree, random forest, and artificial neural network for COVID-19 detection and diagnosis.

Model	Accuracy	Area under the Curve (AUC)	Sensitivity	Specificity	Positive Prediction Value (PPV)	Negative Predictive Value (NPV)
Support Vector Machine (SVM)	88.89%	64.29%	100.00%	88.37%	28.57%	100%
Decision tree	91.11%	71.43%	42.86%	100.00%	100%	90.48%
Random Forest	88.88%	64.29%	28.57%	100.00%	100%	88.37%
Artificial Neural Network	91.11%	83.83%	71.43%	94.74%	71.43%	94.74%

## Data Availability

All data generated or analyzed during this study are included in this published article.
